# Prevalence, correlates and treatment needs of dental caries in patients attending a diabetic clinic in rural southwestern Uganda: a cross-sectional study

**DOI:** 10.1186/s12903-023-03156-y

**Published:** 2023-07-04

**Authors:** Wilfred Arubaku, Deusdedit Tusubira, Frank Ssedyabane, Steffany Chamut, Brittany Anne Seymour, Mark J. Siedner, Vallence Niyonzima, Juliet Nabbanja, Nathan Kakongi, Godfrey Kwizera, Samuel Maling

**Affiliations:** 1 Department of Dental Surgery, Mbarara University of Science and Technology, Mbarara, Uganda; 2 Department of Biochemistry, Mbarara University of Science and Technology, Mbarara, Uganda; 3 Department of Medical Laboratory Science, Mbarara University of Science and Technology, Mbarara, Uganda; 4 Department of Oral Health Policy & Epidemiology, Harvard School of Dental Medicine, Harvard, USA; 5 Department of Medicine, Massachusetts General Hospital, Harvard, USA; 6 Department of Medicine, Harvard Medical School, Harvard, USA; 7 Faculty of Medicine, Mbarara University of Science and Technology, Mbarara, Uganda; 8 Department of Nursing, Mbarara University of Science and Technology, Mbarara, Uganda; 9 Oral health division Ministry of Health, Mbarara, Uganda; 10 Department of Psychiatry, Mbarara University of Science and Technology, P O Box 1410, Mbarara, Uganda

**Keywords:** Dental caries, Diabetes, Treatment needs, Glycemic control, Uganda

## Abstract

**Background:**

Diabetes mellitus is a complex heterogeneous metabolic disorder known to lead to several pathogenic disorders, and has a bidirectional relationship with oral health conditions. This study aimed at estimating the prevalence, treatment needs and correlates of dental caries among adult patients attending a diabetic clinic in Uganda.

**Methods:**

This was a cross-sectional study that used questionnaires to collect data on socio-demographic factors, diabetes history, oral health status, dental health care, dietary factors, lifestyle factors, and dental examination guided by the modified World Health Organization oral health questionnaire for adults.

**Results:**

We enrolled 239 participants, prevalence of dental caries was 71.6%, treatment need was nearly 100%, and mean DMFT was 3.82 (SD = 5.46). Dental caries experience was associated with being widowed.

**Conclusion:**

We found a high prevalence of dental caries experience and large treatment need among our participants. We recommend integration of oral health care into routine diabetic services in rural sub Saharan Africa.

## Introduction

Diabetes mellitus is a leading contributor to the global burden of disease with over 22.9 million new cases reported in 2017 [1]. It is estimated that 8.4% of adults between the ages of 18 and 99 had diabetes in 2017, and that number was expected to climb to 9.9% by 2045 [2]. Furthermore, it is estimated that 14.2 million people aged 20–79 in the sub-Saharan Africa are living with diabetes [3]. Worse still, the prevalence of diabetes in sub-Saharan Africa is rapidly increasing mostly driven by urbanization and changing lifestyles [4, 5]. In 2014, approximately 500,000 Ugandan adults were living with diabetes mellitus (DM), and many more may be living with the disease without their awareness [6]. DM is a heterogeneous metabolic disorder known to lead to several pathogenic disorders, and has a bidirectional relationship with many non-communicable diseases, including oral health conditions [7]. Patients with poorly controlled blood sugar levels have reduced salivary flow rate (xerostomia), resulting in the overgrowth of aciduric bacteria and consequently leading to dental caries [8–10]. Moreover, high plasma glucose levels and the presence of glycated end products in the periodontal tissues [11] can stimulate an inflammatory host response that increases the risk of developing dental caries (tooth decay) [12–15]. However, there are few data from Uganda on the epidemiology of dental caries among adults with DM. In response to this lack of data, we conducted a cross-sectional study with the aim of estimating the prevalence and correlates of dental caries among adults with DM in Uganda.

## Methods

### Study design and setting

We carried out a cross-sectional study at Mbarara Regional Referral Hospital’s (MRRH) diabetes clinic between May – July 2022. The MRRH diabetic clinic has a patient census of approximately 1500 patients. On average, about 80 patients attend the diabetic clinic per week. The MRRH has a catchment area covering 12 districts in southwestern Uganda, as well as, the neighboring countries; Democratic Republic of Congo, Rwanda, and Tanzania.

### Study participants

We recruited adult patients of the MRRH diabetes clinic aged 18 years and above who were previously diagnosed with diabetes attending a routine follow-up appointment and providing written informed consent. We excluded those who were too sick to participate in the study. Participant enrolment was based on convenience sampling of clients according to the way they reported to the clinic during the study period (May – July 2022). When a client declined consent or failed to meet the inclusion criteria we considered the subsequent client.

### Ethical considerations

This study received ethical approval from Mbarara University of Science and Technology-Research and ethics committee (MUST-2022-387) and was registered with Uganda Nation council of science and technology (HS2380ES). Administrative clearance was obtained from the office of the Director of Mbarara regional referral hospital. Written informed consent was obtained from each participant. Those who were unable to read and write in the English language were administered a translated version in the local languages.

### Data collection

We used an interviewer-administered questionnaire with structured open and closed-ended questions to collect data. The questionnaire had five major sections: socio-demographic factors and rural/urban residence, diabetes history, oral health status, dental health care (use of tooth brush, wooden tooth pick, dental floss/ thread, charcoal, chew stick ; dental checkup habits, and use of toothpaste), dietary factors (i.e., consumption of fresh fruits, sugary foods), lifestyle factors (tobacco and alcohol use) and dental examination guided the modified World Health Organization (WHO) oral health questionnaire for adults [16]. Tooth picks are small thin pieces of wood or plastic with one or two pointed ends inserted between teeth to remove detritus after a meal while chew sticks are twigs or roots of certain plants that are used for brushing teeth. Rural urban residence was defined according to the Uganda bureau of statistics criteria. Urban residents defined a city, municipality, gazetted town board or a trading center while rural residence [17]. The questionnaire was administered by a trained research assistant and the dental examination chart was completed by a single well trained dental surgeon with over 20 years’ experience [18]. After completion of the questionnaire, participants were directed to the dental surgeon for a dental examination, as previously described [18]. Disposable dental mirrors and probes under natural light to examine the participant while seated in a reclined position were used. During the examination, the dental surgeon recorded the number of missing, decayed, and filled teeth. Teeth that were traumatized or malformed, missing naturally, or extracted due to trauma, existing periodontal disease, or surgical intervention involving the mouth were excluded. Dental caries were scored using the WHO‘s decayed, filled and missing teeth (DMFT SCORE) index of 1997 [19]. Dental caries experience was defined using a DMFT of ≥ 1. Participants were defined as to have treatment needs if they had decayed or missing teeth l[18]. Respondents with toothache or dental caries were referred to the dental clinic for management, while those with missing teeth were advised to have dentures if necessary.

### Statistical methods

Descriptive analyses on socio-demographic, oral health, rural/urban residence, fasting blood glucose, last dental visit and diabetes characteristics were conducted. These were later stratified by the presence or absence of dental caries experience. Crude prevalence of dental caries and the DMFT index were estimated. Using fitted logistic regression models, correlates of the presence of dental caries were identified. We categorized fasting blood glucose as follows: normal range = 3.9 ---5.6 mmol/L, pre-diabetic_= 5.6–6.9 mmol/L and diabetes ≥ 7 mmol/L we categorized last dental visit as within six months, between six months and one year and beyond one year. The predictor variables of interest included socio-demographic factors (age, sex, education and residence), alcohol and tobacco use, dental health practices (frequency of brushing and dental checkups), and dietary habits (eating fruits, and sugary foods). Variables with a significance of *p < 0.25* in univariable models were included in multivariable models. Analyses were conducted with STATA version 13.

### Sample size

The sample size of 248 participants was determined using the Krejcie & Morgan Table [17] basing on an average of about 350 clients attending the diabetic clinic per month in a study done over 2 months. A population proportion of 0.5 and a confidence interval of 95% were assumed.

## Results

### Population characteristics

The study included 239 participants for analysis with a mean age of 54.4 (SD 13.5) years (Table 1). Approximately three quarters were female (74.5%, 178/239) and half had completed primary school level education (50.6%, 121/239). Participants with less frequent dental check-up (more than 12 months) had significantly higher caries experiences 142/171 (83.0%), p-value = 0.014. Most of the participants regularly used wooden tooth picks 96.7% 231/239, and 93.3% used chew sticks to brush their teeth (223/239).

### Prevalence and characteristics of dental caries experience

Over 70% of participants had dental caries (71.6%, 171/239). As presented in Table 1, there was no statistically significant difference in age groups between those with and without dental caries experience. Among participants with dental caries experience, the majority were females (76.6%, 131/171), however, there was no association between dental caries experience and sex. Furthermore, in terms of fresh fruit consumption, those with dental caries experience (83.6% (143/171) consumed fresh fruits once a week or less, however, there was no association between dental caries experience and fruit consumption. The results also show that 87.7% of participants who did not consume sugar had a caries experience (150/171), however there was no statistically significant difference between those who did/ did not consume sugary foods. The participants who had spent more than 12 months without a routine dental check-up had the highest dental caries experience at 83.0% (142/171). Over 89.5% (153/171) of those who use tooth brush had dental caries experience, and lastly those who brushed with tooth paste and had dental caries experience were 87.1% (149/171). Overall the mean fasting blood sugar levels in our participants was 10.01mmol/L (SD 4.8), this was slightly higher in those with dental caries 10.09 (SD 5.0) as compared to those without 9.8 (SD 4.32). About two thirds (65% 156/239) of our participants had uncontrolled fasting blood glucose levels above 6.9 mm/L although the difference between those with and without caries was not statistically significant.

**Table 1:** Characteristics of participants according to presence or absence of dental caries

	All participants	No Dental caries	Dental caries	p-value
	N = 239	N = 68	N = 171	
		n (%)	n (%)	
**Mean age (SD)**	54.4 (13.5)	52.71 (13.06)	55.01 (13.71)	0.24
**Age group**				
< 35years	16	4/68 (5.9%)	12/171 (7.0%)	1
35-60years	152	44/68 (64.7%)	108/171 (63.2%)	
> 60years	71	20/68 (29.4%)	51/171 (29.8%)	
**Sex**				
Male	61	21/68 (30.9%)	40/171 (23.4%)	0.25
Female	178	47/68 (69.1%)	131/171 (76.6%)	
**Residence**				
Urban	102	23/68 (33.8%)	79/171 (46.2%)	0.085
Rural	137	45/68 (66.2%)	92/171 (53.8%)	
**Marital status**				
Single	20	9/68 (13.2%)	11/171 (6.4%)	0.22
Married	142	42/68 (61.8%)	100/171 (58.5%)	
Divorced	20	5/68 (7.4%)	15/171 (8.8%)	
Widowed	57	12/68 (17.6%)	45/171 (26.3%)	
**Education**				
None	41	11/68 (16.2%)	30/171 (17.5%)	0.031
Primary	121	43/68 (63.2%)	78/171 (45.6%)	
Secondary	46	6/68 (8.8%)	40/171 (23.4%)	
Tertiary	31	8/68 (11.8%)	23/171 (13.5%)	
**Occupation**				
Formally employed	18	6/68 (8.8%)	12/171 (7.0%)	0.47
Self employed	156	47/68 (69.1%)	109/171 (63.7%)	
Not employed	65	15/68 (22.1%)	50/171 (29.2%)	
**Fresh fruit consumption**				
Less than once a week	199	56/68 (82.4%)	143/171 (83.6%)	0.85
More than once a week	40	12/68 (17.6%)	28/171 (16.4%)	
Sugary foods consumption				
No	213	63/68 (92.6%)	150/171 (87.7%)	0.27
Yes	26	5/68 (7.4%)	21/171 (12.3%)	
**Routine dental check up**				
< 6 months	15	1/68 (1.5%)	14/171 (8.2%)	0.014
6–12 months	16	1/68 (1.5%)	15/171 (8.8%)	
> 12 months	208	66/68 (97.1%)	142/171 (83.0%)	
**Reason for last dental care visit**				
Consultation/advise	4	1/11 (9%)	3/90 (3%)	0.76
Pain	51	6/11 (55%)	45/90 (50%)	
Treatment follow up	45	4/11 (36%)	41/90 (46%)	
Routine check up	1	0/11 (0%)	1/90 (1%)	
**Frequency of cleaning teeth**				
Never	2	1/68 (1.5%)	1/171 (0.6%)	0.49
At least once a day	237	67/68 (98.5%)	170/171 (99.4%)	
**Toothbrush usage**				
No	214	61/68 (89.7%)	153/171 (89.5%)	0.96
Yes	25	7/68 (10.3%)	18/171 (10.5%)	
**Wooden toothpick usage**				
No	8	3/68 (4.4%)	5/171 (2.9%)	0.56
Yes	231	65/68 (95.6%)	166/171 (97.1%)	
**Plastic toothpicks usage**				
No	239	68/68 (100.0%)	171/171 (100.0%)	
**Dental floss usage**				
No	239	68/68 (100.0%)	171/171 (100.0%)	
**Charcoal usage**				
No	5	0/68 (0.0%)	5/171 (2.9%)	0.15
Yes	234	68/68 (100.0%)	166/171 (97.1%)	
**Chew stick usage**				
No	16	6/68 (8.8%)	10/171 (5.8%)	0.41
Yes	223	62/68 (91.2%)	161/171 (94.2%)	
**Use of other items/ materials to clean teeth**				
Yes	239	68/68 (100.0%)	171/171 (100.0%)	
		n (%)	n (%)	
**Use of toothpaste**				
No	205	56/68 (82.4%)	149/171 (87.1%)	0.34
Yes	34	12/68 (17.6%)	22/171 (12.9%)	
**Mean fasting blood glucose mmol/L ( SD)**	10.01 (4.8),	9.80 (4.32)	10.09 (5.0)	0.68
**Fasting blood glucose level**				
3.9–5.5	21	2 (2.9%)	19 (11.1%)	0.089
5.6–6.9	62	21 (30.9%)	41 24.0%)	
> 6.9	156	45 (66.2%)	111 (64.9%)	

### Dental caries experience and oral treatment needs

The overall mean DMFT score among study participants was 3.82 (SD 5.46). The total number of decayed teeth was 120, missing teeth 135 and filled teeth, 10 (see Table 2). There was a significant difference (P = 0.015) in the mean DMFT scores across age categories with the age category of 60 years and above having the highest mean DMFT score (5.34 SD 7.49). Female gender had a slightly higher DMFT score (4.04 SD 5.73) however, this was not statistically significant. In this study, those participants who were widowed had the highest DMFT score at 5.09 SD 7.06. In relation to education, participants who did not have formal education had the highest mean DMFT score at 5.19 SD 7.88. Those self-employed had a DMFT Score of 4.08 SD 6.07) and those participants who consumed fruit less than once a week had higher DMFT SCORE of (4.04 SD 5.69), those who consumed sugary foods had a higher DMFT SCORE of 4.92 SD 5.74, for last dental check-, the highest DMFT SCORE was observed in those with last checkup of between 6 and 12 months 9.31 SD 9.13), those who do not use tooth brush had higher DMFT SCORE of 4.24 SD 6.06), higher DMFT SCORE was among those who used toothpaste at 3.83 SD 5.4). Nearly all (98.7%) of the participants had dental caries treatment needs that were not attended to. Our data also revealed that 100% of those with missing teeth had no dentures. In summary, the results show a high mean DMFT score among the study participants and most of the people with DMFT experience are older participants above the age of 60 years and finally the treatment among this group was very high.

**Table 2:** Oral treatment needs among those attending diabetic clinic in southwestern Uganda

	Decayed teeth (DT) (N = 120)	Missing teeth (MT) (N = 135)	Filled teeth (FT) (N = 10)	MeanDMFT SCORE* (N = 239)	Treatment needs**	P value***
	n (%)	n (%)	n (%)	Mean(SD) 3.82 (5.46)	%	
**Age category**						
<35 years	8 (6.67)	9 (6.67)	2 (20.00)	2.38 (3.01)	89.58	0.015
35–60 years	74 (61.67)	84 (62.22)	7 (70.00)	3.26 (4.29)	97.96	
> 60 years	38 (31.67)	42 (31.11)	1 (10.00)	5.34 (7.49)	99.67	
**Sex**						
Male	30 (25.00)	31 (22.96)	0 (0)	3.18 (4.59)	100	0.16
Female	90 (75.00)	104 (77.04)	10 (100.00)	4.04 (5.73)	97.24	
**Residence**						
Urban	56 (46.67)	56 (41.48)	7 (70.00)	3.63 (5.19)	96.17	0.057
Rural	64 (53.33)	79 (58.52 )	3 (30.00)	3.96 (5.67)	99.36	
**Marital Status**						
Single	7 ( 5.83)	8 ( 5.93)	0 (0.00)	2.40 (4.08)	100	0.89
Married	72 (60.00)	80 (59.26)	5 (50.00)	3.41 (4.82)	97.71	
Divorced	12 (10.00 )	9 (6.67)	2 (20.00)	4.55 (5.37)	96.76	
Widowed	29 (24.17)	38 (28.15)	3 (30.00)	5.09 (7.06)	98.13	
**Education**						
None	24 (20.00)	23 (17.04)	0(0.00)	5.19 (7.88)	100	0.41
Primary	53 (44.17)	64 (47.41)	6 (60.00)	3.41 (4.84)	98.18	
Secondary	26 (21.67)	30 (22.22)	2 (20.00)	4.44 (5.69)	95.63	
Tertiary	17 (14.17)	18 (13.33)	2 (20.00)	2.68 (2.57)	98.13	
**Occupation**						
Formal employment	5 (4.17)	11 (8.15)	1 (10.00)	1.94 (2.24)	97.92	0.54
Self employed	80 (66.67)	81 (60.00)	7 (70.00)	4.08 (6.07)	97.22	
Not employed	35 (29.17)	43 (31.85)	2 (20.00)	3.72 (4.39)	99.32	
**Fresh fruit consumption**						
Less than once week	103 (85.83)	113 (83.70)	10 (100.00)	4.04 (5.69)	97.89	0.75
More than Once a week	17 (14.17)	22 (16.30 )	0 (0.00)	2.75 (4.08)	95.46	
**Consumption of sugary foods**						
No	103 (85.83)	120 (88.89)	10 (100.00)	3.69 (5.43)	97.57	0.26
Yes	17 (14.17 )	15 (11.11)	0(0.00)	4.92 (5.74)	100	
**Last dental Check up**						
< 6 months	11 (9.17)	12 (8.89)	1 (10.00)	5.87 (5.94)	98.81	0.68
6 to 12 months	10 (8.33)	14 (10.37)	0(0.00)	9.31 (9.13)	100	
> 12 months	99 (82.50)	109 (80.74)	9 (90.00)	3.25 (4.79)	97.57	
**Reason for last dental check up**						
Consultation/advise	3 (5.45)	1(1.23)	0(0.00)	3 (4.69)	100	0.41
Pain	25 (45.45)	45 (55.56)	1(20.00 )	5.24 (6.02)	100	
Treatment follow up	26 (47.27)	43 (43.21)	4 (80.00)	6.22 (7.24)	99.61	
Routine checkup	1 (1.82 )	0 (0.00)	0 (0.00)	0.0 (0.00)	97.19	
Frequency of cleaning teeth						
Never	1 (0.83)	1 (0.74)	0(0.00)	11 (15.56)	100	0.85
At least once a day	119 (99.17)	134 (99.26)	10 (100.00)	3.76 (5.35)	97.87	
Tooth brush usage						
No	15 (12.50)	15 (11.11)	0(0.00)	3.77 (5.40)	100	0.39
Yes	105 (87.50)	120 (88.89)	10 (100.00)	4.24 (6.06)	97.63	
Use of wooden toothpick						
No	117 (97.50)	131 (97.04)	10 (100.00)	4.63 (7.25)	97.83	0.7
Yes	3 (2.50)	4 (2.96)	0(0.00)	3.79 (5.41)	100	
**Use of plastic toothpicks**						
No	120 (100.0)	120 (100.00)	10 (100.0)	3.82 (5.46)		
Yes	0(0.00)	0(0.00)	0(0.00)	0.0 (0.0)		
**Use of dental floss**						
No	120 (100.0)	135 (100.0)	10 (100.0)	3.82 (5.46)		
Yes	0(0.00)	0(0.00)	0(0.00)	0.0 (0.0)		
**Use of charcoal**						
No	117 (97.50)	130 (96.30)	10 (100.0)	3.86 (5.52)	97.81	0.66
Yes	3 (2.50)	5 (3.70)	0(0.00)	2.20 (0.45)	100	
**Use of chew stick**						
No	112 (93.33)	128 (94.81)	10 (100.0)	**3.85 (5.49)**	97.75	0.53
Yes	8 (6.67)	7 (5.19)	0(0.00)	3.44 (5.23)	100	
**Use of toothpaste**						
No	17 (14.17)	18 (13.33)	2 (20.00)	3.77 (5.90)	94.65	0.14
Yes	103 (85.83)	117 (86.67)	8 (80.00)	3.83 (5.40)	98.36	

*DMFT SCORE, decayed missing, and filled teeth

**Treatment need (decayed + missing teeth / decayed + missing + filled teeth) % i.e. percentage of those with decayed and missing teeth due to caries who did not get treatment by having their teeth filled

***p-values of significance between mean DMFT SCORE according to social participant characteristics calculated using chi-square/ F-testing

n = participants

### Analysis at bivariate and multivariate levels

At bivariate analysis (Table 3) being widowed was associated with 3 times higher odds of dental caries experience (OR = 3.06, 95% CI 1.035–9.095) with a significant P value of 0.043. Being widowed remained significantly (P value 0.014) associated with dental caries experience at multivariate level (Table 3) with adjusted Odds ratio (AOR = 4.854, 95% CI 1.356–17.378). Residing in a rural setting was associated with reduced likelihood of experiencing dental caries (OR = 0.602, CI 0.335–1.08 P value 0.089). A similar observation was made at multivariate analysis (AOR = 0.514, CI 0.261–1.014 P value 0.049).

**Table 3:** Logistic regression analysis

CATEGORY	Bivariate analysis			Multivariate analysis		
	OR	CI	*P value*	AOR	CI	*P value*
**Sex**						
Male	1					
Female	1.427	0.766–2.660	0.262	1.19	0.596–2.491	0.619
**Residence**						
Urban	1					
Rural	0.602	0.335–1.08	0.089	0.514	0.261–1.014	0.049
**Marital status**						
Single	1					
Married	1.967	0.759–5.096	0.163	2.848	0.961–8.447	0.059
Divorced	2.454	0.642–9.391	0.19	3.437	0.781–15.137	0.103
Widowed	3.068	1.035–9.098	0.043*	4.854	1.356–17.378	0.014*
**Education**						
None	1					
Primary	0.665	0.303–1.458	0.308	0.594	0.249–1.413	0.239
Secondary	2.444	0.812–7.355	0.112	2.573	0.769–8.596	0.125
Tertiary	1.1	0.382–3.166	0.86	0.996	0.756–7.650	0.994
**Consumption of sugary foods**						
No	1					
Yes	1.748	0.631–4.843	0.283	1.419	0.756–7.651	0.134
**Last dental check up**						
< 6 months	1					
6 to 12 months	1.071	0.061–18.819	0.962	1.37	0.072–26.083	0.829
> 12 months	0.156	0.021–1.211	0.076	0.142	0.017–1.157	0.067
**Toothbrush usage**						
No	1					
Yes	0.992	0.394–2.495	0.986	0.82	0.291–2.314	0.722

## Discussion

In the present study we investigated the prevalence, correlates and treatment needs of dental caries among adults with diabetes mellitus in Uganda. We found a high prevalence of dental caries among people attending the diabetic clinic in south western Uganda. The most common type was missing component of DMFT and majority of the study participants had not had a dental check-up in the past 12 months. Most of those with dental caries were middle aged (35–60 years) and resided in rural areas. Being widowed was associated with higher dental caries experience, and the results remained highly significant at bivariate and multivariate analysis. Results from this study reveal a glaring gap in oral health prevention needs in patients attending diabetes care in rural south western Uganda, with nearly 100% of participants having a treatment need of either missing or decayed teeth. Majority of the participants had not gone for a dental checkup in the previous one year, despite their dental caries experience which observation has been reported in African and middle eastern regions [20].

The high prevalence of dental caries experience in our study participants is comparable to results from other studies. Comparably high prevalence of dental caries among diabetic patients was found by Malvania et al. [21], and Varughese et al. [22] in India, Barylo et al[23] in Ukraine, Lin et al. [24] in USA and Almusawi et al[25] and Guinan et al[26] in Africa. Additionally we found that participants with fasting blood glucose levels of more than 6.9mmol/L had higher levels of dental caries experience which is in agreement with previous studies [21–26]. This could be a result of the high glucose levels in the saliva promoting bacterial growth [13, 21, 27]. Further more people living with diabetes are prone to increased intake of cariogenic food [28, 29] with exacerbates the chances of developing tooth decay and gum disease. A study from Cameroon reported a significantly lower dental caries prevalence of 21.5% in patients with diabetes [30]. This could be true if those living with diabetes know more about what to eat and thus minimize intake of cariogenic foods especially sugar [21, 31]. On the other hand, findings from a study in Tunisia suggest that glycemic control is not associated with dental caries experience [32]. Similar findings were described in a review by Taylor et al. [33].

We found a DMFT score of 3.82 among our participants. This is average and is comparable with results from a similar population of diabetic patients in Ivory Coast [26]. However, a higher DMFT was found by Barylo et al. [23] in Ukraine and in a hospital-based cross-sectional study carried out by Malvania et al. [21] in India. Participants whose last dental checkup was more than 12 months had the lowest DMFT which could mean that they only sought care when they had a dental problem. These finding are in agreement with general observation that regular dental visits improves oral health outcomes [34, 35]. Older age of our participants was associated with a higher DMFT scores. This is in line with other observations demonstrating that dental caries experience is high in older adults [36–38].

Our results also show that the prevalence of dental caries experience was three times higher in female participants although the difference was not statistically significant. This is similar to other studies as reviewed by Teshome et al. [39]. We found that participants who consumed less fruits had more dental caries, however the difference was not statistically significant. This could be partially explained by the protective role of xylitol, a common constituent of fruit that has been shown to reduce dental caries in adults and children [40, 41]. Other fruit extract have also shown similar protective properties [41]. The role of fruit and fruit extract in protecting against dental caries in patients with diabetes may require further investigation especially in areas of low resource settings.

The treatment need among our participants was very high with nearly all participants having either missing or decayed teeth. Very high treatment needs have also been described elsewhere such as by Teshome et al. [42] and Bogale et al. [43] in Ethiopia, and by Van Wyk et al. in south Africa [44], High treatment needs may partly result from lack of awareness on the potential consequences of dental caries and tooth loss among our participants [45].

Our study had limitations: We relied on fasting glucose sugar levels to assess glycemic control instead of glycated Hemoglobin because fasting glucose sugar level was the only routinely done test among our participants. In addition, records categorizing patients as type I and II diabetes were not available hence could not analyze for differences among participants in this regard.

Conclusion and recommendations: we observed a high prevalence of dental caries in our study population, and an extremely high treatment need of dental caries in study population. In summary, only being widowed was associated with dental caries prevalence. In view of our results we therefore recommend that oral health services be integrated into diabetes care.

## Data Availability

The datasets used and/or analyzed during the current study are available from the corresponding author SM on reasonable request. All data generated or analyzed during this study are included in this published article.

## References

[1] Lin X, Xu Y, Pan X, Xu J, Ding Y, Sun X, Song X, Ren Y, Shan PF (2020). Global, regional, and national burden and trend of diabetes in 195 countries and territories: an analysis from 1990 to 2025. Sci Rep.

[2] Cho NH, Shaw JE, Karuranga S, Huang Y, da Rocha Fernandes JD, Ohlrogge AW, Malanda B (2018). IDF Diabetes Atlas: global estimates of diabetes prevalence for 2017 and projections for 2045. Diabetes Res Clin Pract.

[3] Assah F, Mbanya JC. Diabetes in Sub-Saharan Africa. In: Diabetes Mellitus in Developing Countries and Underserved Communities. edn. Edited by Dagogo-Jack S. Cham: Springer International Publishing; 2017: 33–48.

[4] Mbanya JCN, Motala AA, Sobngwi E, Assah FK, Enoru ST (2010). Diabetes in sub-saharan Africa. The Lancet.

[5] Hall V, Thomsen RW, Henriksen O, Lohse N (2011). Diabetes in sub Saharan Africa 1999–2011: epidemiology and public health implications. A systematic review. BMC Public Health.

[6] Bahendeka S, Wesonga R, Mutungi G, Muwonge J, Neema S, Guwatudde D (2016). Prevalence and correlates of diabetes mellitus in Uganda: a population-based national survey. Tropical Med Int Health.

[7] Sanz M, Ceriello A, Buysschaert M, Chapple I, Demmer RT, Graziani F, Herrera D, Jepsen S, Lione L, Madianos P (2018). Scientific evidence on the links between periodontal diseases and diabetes: Consensus report and guidelines of the joint workshop on periodontal diseases and diabetes by the International Diabetes Federation and the European Federation of Periodontology. Diabetes Res Clin Pract.

[8] Latti BR, Kalburge JV, Birajdar SB, Latti RG (2018). Evaluation of relationship between dental caries, diabetes mellitus and oral microbiota in diabetics. J oral maxillofacial pathology: JOMFP.

[9] Soell M, Hassan M, Miliauskaite A, Haïkel Y, Selimovic D (2007). The oral cavity of elderly patients in diabetes. Diabetes Metab.

[10] Laouali N, El Fatouhi D, Aguayo G, Balkau B, Boutron-Ruault M-C, Bonnet F, Fagherazzi G (2021). Type 2 diabetes and its characteristics are associated with poor oral health: findings from 60,590 senior women from the E3N study. BMC Oral Health.

[11] Graves DT, Ding Z, Yang Y (2020). The impact of diabetes on periodontal diseases. Periodontol 2000.

[12] Goodson JM, Hartman M-L, Shi P, Hasturk H, Yaskell T, Vargas J, Song X, Cugini M, Barake R, Alsmadi O (2017). The salivary microbiome is altered in the presence of a high salivary glucose concentration. PLoS ONE.

[13] Yang Y, Liu S, Wang Y, Wang Z, Ding W, Sun X, He K, Feng Q, Zhang X (2020). Changes of saliva microbiota in the onset and after the treatment of diabetes in patients with periodontitis. Aging.

[14] Wei Y-S, Hsiao Y-C, Su G-W, Chang Y-R, Lin H-P, Wang Y-S, Tsai Y-T, Liao E-C, Chen H-Y, Chou H-C (2020). Identification of hyperglycemia-associated microbiota alterations in saliva and gingival sulcus. Arch Biochem Biophys.

[15] Almusawi MA, Gosadi I, Abidia R, Almasawi M, Alrashood ST, Ekhzaimy A, Alhomida AS, Khan HA (2020). Association between salivary factors and cariogenic bacteria in type-2 diabetes patients. J King Saud Univ - Sci.

[16] World Health Organization. Oral health surveys: basic methods. World Health Organization; 2013.

[17] Uganda Bureau of Statistics. : Uganda National Population and Housing Census 2014. Provisional Results.

[18] Arubaku W, Kwizera G, Tusubira D, Kanyesigye M, Chamut S, Seymour BA, Siedner MJ, Niyonzima V, Najjuma JN, Maling S (2022). Prevalence, correlates and treatment needs of dental caries among people on antiretroviral therapy in Uganda: a cross sectional study. BMC Oral Health.

[19] Organization WH. Oral health surveys: basic methods. World Health Organization; 2013.

[20] Abid A, Maatouk F, Berrezouga L, Azodo C, Uti O, El-Shamy H, Oginni A (2015). Prevalence and severity of oral diseases in the Africa and Middle East Region. Adv Dent Res.

[21] Malvania EA, Sheth SA, Sharma AS, Mansuri S, Shaikh F, Sahani S (2016). Dental caries prevalence among type II diabetic and nondiabetic adults attending a hospital. J Int Soc Prev Community Dentistry.

[22] Varughese A, Kavitha R, Sravan Kumar Y, Venkitachalam R, Menon AS, Francis PT, Haridas K (2022). Prevalence and severity of coronal and radicular caries among patients with type 2 diabetes mellitus: a cross sectional study. Medical journal. Armed Forces India.

[23] Barylo ОS, Kanishyna Т, Shkilniak LI. The effects of diabetes mellitus on patients’ oral health. Wiadomosci lekarskie (Warsaw, Poland: 1960) 2018, 71(5):1026–1031. 30176635

[24] Lin BP, Taylor GW, Allen DJ, Ship JA (1999). Dental caries in older adults with diabetes mellitus. Spec Care Dentist.

[25] Almusawi M, Gosadi I, Abidia R, Almasawi M, Khan H (2018). Potential risk factors for dental caries in type 2 diabetic patients. Int J Dental Hygiene.

[26] Open Journal of Epidemiology 2018, Vol.08No.04:13.

[27] Goodson JM, Hartman ML, Shi P, Hasturk H, Yaskell T, Vargas J, Song X, Cugini M, Barake R, Alsmadi O (2017). The salivary microbiome is altered in the presence of a high salivary glucose concentration. PLoS ONE.

[28] Mishra VK. Association between Dental Caries and Type 2 Diabetes Mellitus among Kanpur Population.

[29] Shiferaw A, Alem G, Tsehay M, Kibret GD. Dental caries and associated factors among diabetic and nondiabetic adult patients attending Bichena Primary Hospital’s Outpatient Department. Front Oral Health 2022, 3. 10.3389/froh.2022.938405 PMC9666893 36407659

[30] Bissong M, Azodo CC, Agbor MA, Nkuo-Akenji T, Fon PN. Oral health status of diabetes mellitus patients in Southwest Cameroon. Odonto-stomatologie tropicale = tropical dental journal 2015, 38(150):49–57. 26934773

[31] Singh I, Singh P, Singh A, Singh T, Kour R (2016). Diabetes an inducing factor for dental caries: a case control analysis in Jammu. J Int Soc Prev Community Dentistry.

[32] Sebai I, Temessek A, Chelly A, Harrabi T, Ben Mami F (2019). Assessment of oral health status among uncontrolled diabetic mellitus patients in Tunisia. Tunis Med.

[33] Taylor GW, Manz MC, Borgnakke WS. Diabetes, periodontal diseases, dental caries, and tooth loss: a review of the literature. Compendium of continuing education in dentistry (Jamesburg, NJ: 1995) 2004, 25(3):179–184, 186 – 178, 190; quiz 192. 15641324

[34] Åstrøm AN, Ekback G, Ordell S, Gulcan F (2018). Changes in oral health-related quality of life (OHRQoL) related to long-term utilization of dental care among older people. Acta Odontol Scand.

[35] Crocombe LA (2015). Long-term routine dental attendance is important for older adults. J Evid Based Dent Pract.

[36] Frencken J. Caries Epidemiology and Its Challenges. Monographs in oral science 2018, 27:11–23. 10.1159/000487827 29794449

[37] Saunders RH, Meyerowitz C (2005). Dental caries in older adults. Dent Clin.

[38] López R, Smith PC, Göstemeyer G, Schwendicke F (2017). Ageing, dental caries and periodontal diseases. J Clin Periodontol.

[39] Teshome A, Muche A, Girma B (2021). Prevalence of dental caries and associated factors in East Africa, 2000–2020: systematic review and meta-analysis. Front Public Health.

[40] Honkala S, Runnel R, Saag M, Olak J, Nõmmela R, Russak S, Mäkinen PL, Vahlberg T, Falony G, Mäkinen K (2014). Effect of erythritol and xylitol on dental caries prevention in children. Caries Res.

[41] Nayak PA, Nayak UA, Khandelwal V (2014). The effect of xylitol on dental caries and oral flora. Clin Cosmet Invest dentistry.

[42] Teshome A, Andualem G. Dental Caries and Associated factors among patients attending the University of Gondar Comprehensive Hospital Dental Clinic, North West Ethiopia: A Hospital-Based cross-sectional study. 2020, 12:191–8. 10.2147/CCIDE.S247179 PMC7250298 32547246

[43] Bogale B, Engida F, Hanlon C, Prince MJ, Gallagher JE (2021). Dental caries experience and associated factors in adults: a cross-sectional community survey within Ethiopia. BMC Public Health.

[44] Van Wyk C, Van Wyk PJ (2010). Trends in dental caries prevalence, severity and unmet treatment need levels in South Africa between 1983 and 2002: scientific. South Afr Dent J.

[45] Nazir MA, AlGhamdi L, AlKadi M, AlBeajan N, AlRashoudi L, AlHussan M (2018). The burden of diabetes, its oral complications and their Prevention and Management. Open access Macedonian journal of medical sciences.

